# Pulmonary mucinous cystadenoma complicated with infection: A rare case report

**DOI:** 10.1097/MD.0000000000026906

**Published:** 2021-08-13

**Authors:** Zhou-Ye Luo, Xun-Ze Shen, Fang Liu, Chen Lin

**Affiliations:** a PET/CT Center, Shaoxing People's Hospital (Shaoxing Hospital, Zhejiang University School of Medicine), Shaoxing, Zhejiang Province, China; b Department of Pathology, Shaoxing People's Hospital (Shaoxing Hospital, Zhejiang University School of Medicine), Shaoxing, Zhejiang Province, China.

**Keywords:** imaging., infection, lung, mucinous cystadenoma, tumor

## Abstract

**Rationale:**

Mucinous cystadenoma is a benign tumor that is commonly found in the pancreas, ovaries, or appendix, but is rarely encountered in the lungs. Worldwide, only a few reported cases of these tumors originate in the lungs. Herein, we analyzed the imaging features of a case of pulmonary mucinous cystadenoma (PMCA). To the best of our knowledge, this is the first reported case of PMCA complicated by significant infection.

**Patient concerns:**

A 57-year-old man was admitted to our hospital with blood in sputum for more than 2 months. Serum laboratory examination showed significantly elevated leukocyte and tumor marker, carcinoembryonic antigen. Enhanced thoracic computed tomography and whole-body positron emission tomography/computed tomography showed a cystic-solid ill-defined mass in the right upper lung.

**Diagnosis:**

The tumor was considered malignant, both clinically and radiologically.

**Interventions:**

The patient underwent right upper lobe tumor resection and mediastinal lymph node dissection.

**Outcomes:**

Postoperative specimen pathology was diagnosed as PMCA with infection. The patient was not administered any further treatment. The patient was alive without any recurrence or metastasis of the tumor after 2 years of follow-up.

**Lessons:**

Preoperative diagnosis of PMCA with atypical imaging and clinical manifestations is extremely difficult. This is the first reported case of PMCA complicated by a significant infection that was misdiagnosed preoperatively as a malignancy.

## 1 Introduction

Mucinous cystadenoma of the lung is a rare benign tumor that has rarely been reported.^[1–3]^ To date, fewer than 20 cases of pulmonary mucinous cystadenoma (PMCA) have been reported in the English literature worldwide.^[3]^ They were located in the periphery of any lobe of the lung, with a preference toward the right side.^[3,4]^ The tumor is usually unilocular and filled with mucus.^[1–5]^ The cyst wall was lined with mucinous epithelium with varying degrees of atypia.^[3–5]^ Typical imaging features of this tumor tend to be well-demarcated thin-walled singular cystic masses in the periphery of the lung parenchyma.^[4,5]^ When the mass is large, it can cause distortion of the surrounding tissue, resulting in inflammation and atelectasis.^[3]^ Herein, we report a rare case of PMCA complicated with infection, which was misdiagnosed as a malignancy on preoperative computed tomography (CT), positron emission tomography/CT (PET/CT), and clinical findings.

## 2 Case presentation

A 57-year-old male patient was referred to our hospital because of blood in the sputum for more than 2 months and a lung mass found on a plain CT scan in a local hospital. The patient had a small amount of white mucous sputum with bright red blood filaments, without chest pain or abnormal body temperature.

The patient's laboratory examination in our hospital showed that the white blood cell count increased to 13.59 × 10^9^/L in the blood routine, and neutrophils accounted for 90.6%. In the male tumor markers combination (14 items), carcinoembryonic antigen (CEA) serum level increased to 19.76 ng/mL (normal range, 0–5 ng/mL), and the other markers were within the normal range.

**Figure 1:**
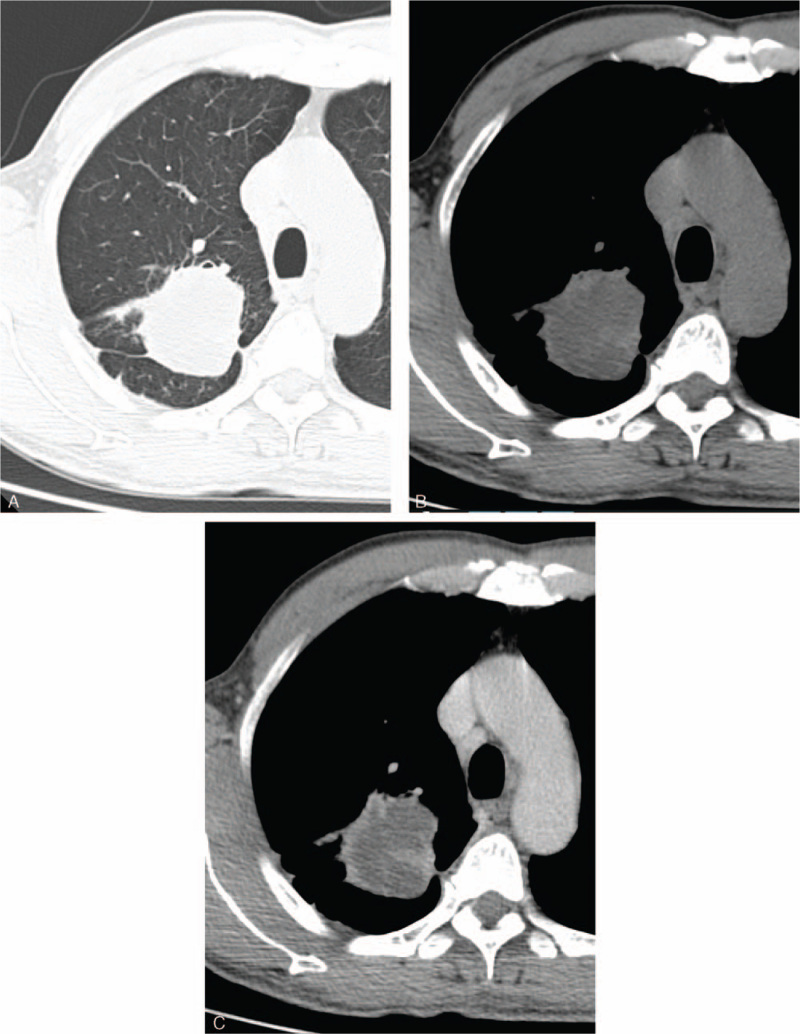
Chest contrast-enhanced CT revealed an analogous round mass in the posterior segment of the right upper lobe with heterogeneous enhancement, and extensive central liquefaction, a few striped shadows around the tumor. (A) CT plain scan of lung window; (B) CT plain scan of the mediastinal window; and (C) Contrast-enhanced CT scan of the mediastinal window. CT = computed tomography.

Chest contrast-enhanced CT revealed a 53 × 46 mm mass in the posterior segment of the right upper lobe with heterogeneous enhancement, extensive central liquefaction, a few striped shadows around the tumor, and multiple enlarged mediastinal lymph nodes (Fig. 1A to C). Lung cancer with a small amount of peripheral obstructive inflammation is considered and further examination is recommended to assist in the diagnosis.

**Figure 2:**
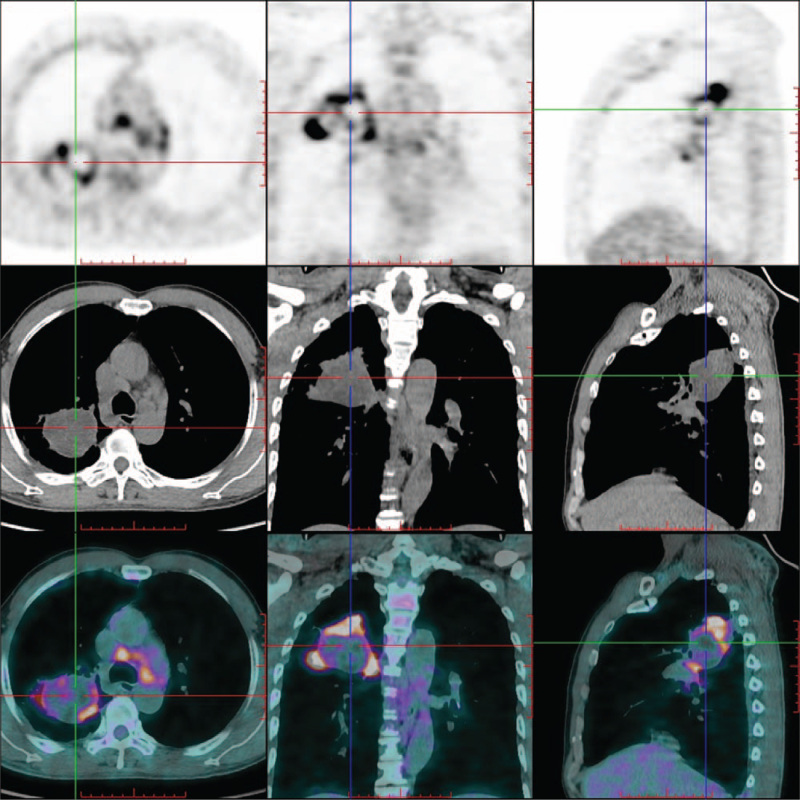
The whole body 3D PET/CT images showed a cystic-solid mass in the right thorax with irregular margins. The thickness of the cyst wall was not uniform, the cystic wall showed significantly increased FDG uptake, but the central cystic component was not accompanied by FDG uptake. Several enlarged lymph nodes with increased FDG uptake could be seen in the mediastinum. (top row: PET scan images; middle row: unenhanced CT scan images; and bottom row: PET and CT fusion images). FDG = fluorodeoxyglucose, PET/CT = positron emission tomography/computed tomography.

Whole-body PET/CT showed a round cystic-solid mass in the posterior segment of the right upper lobe, with irregular margins. The thickness of the cyst walls was not uniform. The cystic wall showed significantly increased fluorodeoxyglucose (FDG) uptake (maximum standardized uptake value was approximately 8.70), but the central cystic component was not accompanied by FDG uptake. Several enlarged lymph nodes with increased FDG uptake were observed in the right hilum and mediastinum, and the maximum standardized uptake value was approximately 4.82 (Fig. 2).

The doctors considered the possibility of a malignant tumor in the right upper lobe with multiple lymph node metastases in the right hilum and mediastinum, and suggested a needle biopsy at the site with a high metabolism and more solid components at the upper edge of the tumor. Chronic mucosal inflammation with interstitial fibrous hyperplasia and mucus lake formation was observed in the biopsy specimen of the right upper lung tumor. Very few atypical cells were observed in the mucus, and no evidence of cancer was found.

After a comprehensive analysis of imaging examination and laboratory examination, the clinician still believed that the patient's right upper lung mass had the possibility of malignancy, so the patient underwent a right upper lobe resection plus mediastinal lymph node dissection. During the operation, a 5 × 5 cm mass was observed in the right upper lung, containing a yellow jelly-like substance, and lymph node enlargement in groups 2, 4, 7, 10, 11, and 12.

**Figure 3:**
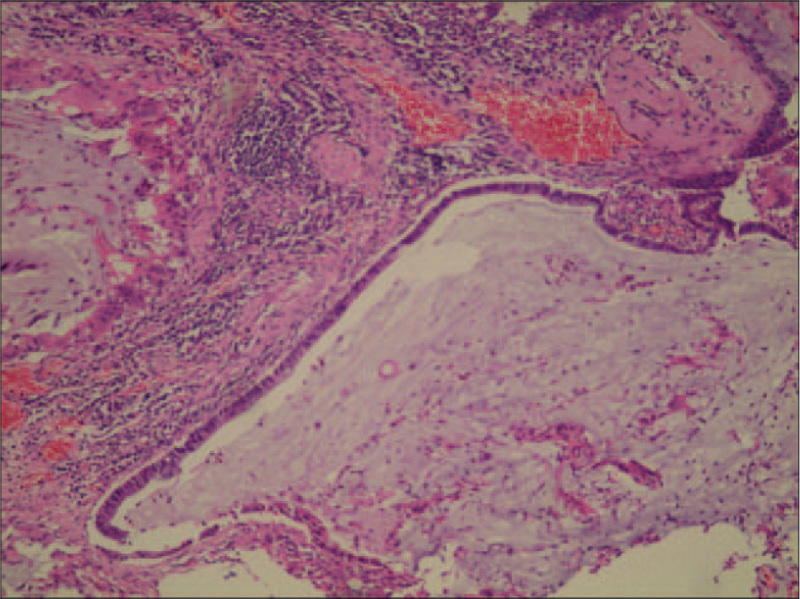
Histological examination showed that the cyst wall is lined with single columnar epithelium and the cytoplasm contains mucus. Epithelial shedding in most areas. There were many lymphocytes and plasma cells in the cyst wall, accompanied by hemorrhage and histiocytic reaction, and the formation of multinucleated giant cells (hematoxylin and eosin staining, ×100).

Pathological examination of the surgically resected specimen showed inflammatory fibroplasia and inflammatory cell infiltration in the right upper lung mass, and a multinucleated giant cell reaction was observed locally. Mucinous lake formation was observed in the larger tissue, and a small amount of mucinous columnar epithelial cell proliferation was observed around the mucinous lake, which was considered to be a mucinous neoplastic lesion (Fig. 3). Immunophenotyping revealed positive staining for CK7, Ki-67 (5%), and CDX2, but negative staining for CK20, NapsinA, thyroid transcription factor-1 (TTF-1), CK5/6, P53, and Villin. The final pathological diagnosis was mucinous cystadenoma of the right upper lung with inflammatory changes in the surrounding lung tissue and multiple chronic inflammations of the mediastinal lymph nodes.

The patient recovered well and was not receiving any further treatment. One year later, the male tumor index combination (14 items) was reexamined, and the results were all within the normal range with a CEA value of 2.31. The patient was alive without any recurrence or metastasis of the tumor after 2 years of follow-up.

Ethical approval was not required because this was a case report of the patient's clinical information. Written informed consent was obtained from the patient for the publication of this case report and accompanying images.

## 3 Discussion

Mucinous cystadenoma is defined as “a localized cystic mass filled with mucin and surrounded by a fibrous wall lined by well-differentiated columnar mucinous epithelium.”^[1]^ Mucinous cystadenoma is a benign tumor that is commonly found in the pancreas, ovaries, or appendix. Worldwide, only a few reported cases of these tumors originate in the lungs. PMCA was first described by Gower in 1978 as “an unusual mucous cyst in the lung.”^[2]^ The age of patients with PMCA ranges from 32 to 75 years (median, 61 years), and the tumor seems to be more common in women. They were located in the periphery of any lobe of the lung, with a preference toward the right side. The median size was 5 cm (range, 0.8–15 cm).^[3]^ Although our patient was male, he was nearly 60 years old, with a mass size of approximately 4.6 × 5.3 cm, also located in the right lung, which was generally consistent with the literature reports.

The histology of this neoplasm is characterized by a localized cystic mass and benign proliferation of mucus-producing epithelial cells.^[2–4]^ Immunohistochemical (IHC) studies usually show positivity for pan-cytokeratin (CK) and, in some cases, CEA, surfactant-associated protein A, and negative stain for TTF-1. These IHC features suggest that the tumor is derived from the non-alveolar epithelium.^[3,4]^ A positive CEA indicates that the tumor has a local malignancy or is prone to recurrence after surgery. IHC examination of our patient specimen showed positive CK7 and negative TTF-1 expression, which was consistent with the literature reports. Although CEA staining was not performed, the patient's serum CEA level was significantly elevated preoperatively, suggesting that the tumor had malignant potential.

Radiographically PMCA appears as a well-defined, homogeneous lesion.^[3,4]^ PMCA is usually well-circumscribed and homogeneous on CT, and the cyst wall is usually thin. Inflammation and adjacent atelectasis only seem to be present after a certain enlargement of the lesion due to compression and distortion of the surrounding tissue.^[2–5]^ On FDG PET/CT, the tumor usually shows no abnormal uptake due to its thin wall and mucus-filled interior.^[4]^ In our case, the findings on CT and PET/CT were different from those reported in the literature. The interior of the tumor was mainly cystic, but there were still uneven high-density lesions, uneven wall thickening, and obviously abnormal FDG uptake, which led to misdiagnosis as a malignant tumor, but histopathologically confirmed as mucinous cystadenoma with inflammatory changes.

Although PMCA is a benign tumor, it may have some degree of malignant potential, and early and complete resection with a sufficient margin is required for the treatment.^[4]^ Sometimes, depending on the local condition, resection of these lesions can also be minimally invasive.^[6]^ Further follow-up is usually required based on pathological findings.^[7]^ The prognosis for these patients is usually good, with recurrence being rare and metastases from malignant lesions being exceptional.^[3,6,7]^ Matsuo et al reported a case of PMCA with tumor recurrence 20 years after surgery, which was the only case of recurrence reported in the literature.^[7]^ Matsuo et al reported a case of pulmonary mucinous cystadenocarcinoma arising from PMCA,^[7]^ and Davison et al reported a case of adenocarcinoma arising in a mucinous cystadenoma of the lung.^[8]^ In our patient with PMCA, the serum CEA level was as high as 19.76 ng/mL before surgery, and it was reduced to 2.31 ng/mL 1 year after surgery, which also indicated that this tumor had a certain degree of malignancy and required regular follow-up. KRAS mutations, which are typical for mucinous carcinomas of the lung, may also be a mechanism for the development of mucinous cystadenocarcinoma arising from PCMA.^[3,4]^

In conclusion, a mucinous cystadenoma is usually a grossly well-demarcated peripheral cyst filled with gelatinous mucin, but this benign tumor may have some degree of malignant potential. We encountered an extremely rare case of PCMA associated with infection. The CT and FDG PET/CT imaging findings of this patient were relatively special, and the serum CEA level was also significantly increased. The clinical diagnosis was very difficult, and complete resection of the tumor was necessary.

## Author contributions

**Conceptualization:** Xun-Ze Shen (Shen XZ).

**Formal analysis:** Zhou-Ye Luo (Luo ZY).

**Resources:** Fang Liu (Liu F).

**Writing – original draft:** Xun-Ze Shen (Shen XZ), Zhou-Ye Luo (Luo ZY), Chen Lin (Lin C).

**Writing – review & editing:** Xun-Ze Shen (Shen XZ).
